# Emerging Resistance to Empiric Antimicrobial Regimens for Pediatric Bloodstream Infections in Malawi (1998–2017)

**DOI:** 10.1093/cid/ciy834

**Published:** 2018-10-01

**Authors:** Pui-Ying Iroh Tam, Patrick Musicha, Kondwani Kawaza, Jenifer Cornick, Brigitte Denis, Bridget Freyne, Dean Everett, Queen Dube, Neil French, Nicholas Feasey, Robert Heyderman

**Affiliations:** 1 Malawi-Liverpool Wellcome Trust, Institute of Infection and Global Health, University of Liverpool, United Kingdom; 2 Liverpool School of Tropical Medicine, United Kingdom; 3 University of Malawi College of Medicine, Blantyre; 4 Institute of Infection and Global Health, University of Liverpool, United Kingdom; 5 The Queens Medical Research Institute, University of Edinburgh, United Kingdom; 6 University College London, United Kingdom

**Keywords:** antimicrobial resistance, pediatric, neonatal, sepsis, Gram negative

## Abstract

**Background:**

The adequacy of the World Health Organization’s Integrated Management of Childhood Illness (IMCI) antimicrobial guidelines for the treatment of suspected severe bacterial infections is dependent on a low prevalence of antimicrobial resistance (AMR). We describe trends in etiologies and susceptibility patterns of bloodstream infections (BSI) in hospitalized children in Malawi.

**Methods:**

We determined the change in the population-based incidence of BSI in children admitted to Queen Elizabeth Central Hospital, Blantyre, Malawi (1998–2017). AMR profiles were assessed by the disc diffusion method, and trends over time were evaluated.

**Results:**

A total 89643 pediatric blood cultures were performed, and 10621 pathogens were included in the analysis. Estimated minimum incidence rates of BSI for those ≤5 years of age fell from a peak of 11.4 per 1000 persons in 2002 to 3.4 per 1000 persons in 2017. Over 2 decades, the resistance of Gram-negative pathogens to all empiric, first-line antimicrobials (ampicillin/penicillin, gentamicin, ceftriaxone) among children ≤5 years increased from 3.4% to 30.2% (*P* < .001). Among those ≤60 days, AMR to all first-line antimicrobials increased from 7.0% to 67.7% (*P* < .001). Among children ≤5 years, *Klebsiella* spp. resistance to all first-line antimicrobial regimens increased from 5.9% to 93.7% (*P* < .001).

**Conclusions:**

The incidence of BSI among hospitalized children has decreased substantially over the last 20 years, although gains have been offset by increases in Gram-negative pathogens’ resistance to all empiric first-line antimicrobials. There is an urgent need to address the broader challenge of adapting IMCI guidelines to the local setting in the face of rapidly-expanding AMR in childhood BSI.

## Teaser

Incidences of bloodstream infections in Malawian children declined significantly over 2 decades, but resistance of Gram-negative pathogens to empiric, first-line antimicrobials increased from 3.4% to 30.2% for children ≤5 years and 7.0% to 67.7% for young infants ≤60 days.

Sepsis accounted for half a million child deaths globally in 2000, and had the lowest decline of all the top 7 causes of death in this population by 2015, with only a 25% decrease compared to other leading causes, such as pneumonia (47%), diarrhea (57%) and measles (85%) [1]. In 2015, sepsis and other infectious conditions of the newborn accounted for 7% of all deaths worldwide among children under 5 years of age [1]. Empiric, first-line antimicrobial treatment for sick infants in the World Health Organization’s (WHO’s) Integrated Management of Childhood Illness (IMCI) guidelines consists of penicillin/ampicillin with gentamicin, or ceftriaxone (as is practiced in Malawi). At our center in Malawi, ceftriaxone has been available since 2001, was introduced as an empiric antibiotic for neonatal meningitis in 2009 [2], and has been used as the first-line treatment for suspected neonatal sepsis since 2013 [3]. Pediatric departmental guidelines recommend the use of penicillin and gentamicin for suspected sepsis or ceftriaxone for suspected meningitis, typhoid fever, and non-typhoidal *Salmonella* (NTS). These recommendations are based on sparse data from low- and middle-income countries (LMICs), and are intended to be adjusted to local susceptibility patterns so that “appropriate therapy [can be given] for an identified bacterial cause” [4]. This can be challenging in LMICs, where a paucity of diagnostic microbiology facilities [5] limits the ability of clinicians to optimize empiric therapy.

Child mortality in Malawi has been falling in the last 20 years as a consequence of multiple interventions, including better nutrition, new vaccines (including the *H. influenzae* type B conjugate vaccine in 2002 and *S. pneumoniae* conjugate vaccine in 2011), rapid roll-out of human immunodeficiency virus prevention and care, and improvements in malaria control [6–11]. A growing problem has been increasing rates of antimicrobial resistance (AMR) [12, 13], which may be caused and compounded by inappropriate prescribing, under-dosing, and nonadherence to guidelines [14, 15]. There is a scarcity of high-quality bloodstream-infection (BSI) surveillance data to inform policy change. We have previously documented the expansion of extended-spectrum beta-lactamase (ESBL) and fluoroquinolone resistance among common Gram-negative pathogens, as well as the emergence of methicillin-resistant *Staphylococcus aureus* in BSI [12], but did not explore this in detail in the population ≤5 years, where the impact may be greatest. This study reviews the pediatric surveillance data collected over 2 decades at an urban district hospital and tertiary referral center in Malawi. We describe trends in the etiology and the prevalence of AMR amongst BSI isolates cultured from hospitalized children. Finally, we assess the adequacy of current IMCI guidelines in this setting.

## METHODS

### Setting

Queen Elizabeth Central Hospital (QECH) in Blantyre, Malawi, is a 1250-bed, government-funded teaching hospital providing free medical care, and is the main teaching hospital of the University of Malawi College of Medicine. It is the referral center for the southern half of the country and serves as the district hospital for the urban Blantyre area (estimated population 920000 in 2016). The pediatric department admits 20000–30000 children a year, with 65000–80000 seen annually in the pediatric Accident and Emergency unit. Total numbers of pediatric hospital admissions have remained broadly constant over the study period (Supplementary Table 1). Similarly, the neonatal unit admits 3500 neonates a year, and these numbers have remained consistent over the past decade, since admission data were first collected.

### Study Design

We reviewed blood culture data collected at QECH over 20 consecutive years (1998–2017; Supplementary Figure 1). Blood culture sampling in children was obtained from any pediatric patient with a clinical suspicion of sepsis, severe sepsis, or septic shock [16]; with a non-focal febrile illness and a negative test for malaria; or who remained febrile despite empiric antimicrobial treatment. Sampling followed set departmental guidelines, which have remained unchanged over the period of surveillance. Repeat blood cultures were not routinely done in the setting of a positive result. Precise individual- or population-based antimicrobial usage data for the surveillance period were not available. An analysis of microbiological surveillance data was approved by the University of Malawi College of Medicine Research Ethics Committee (P.08/14/1614).

### Microbiology

Routine, quality-assured, diagnostic blood culture services have been provided for children admitted to QECH by the Malawi-Liverpool-Wellcome Trust Clinical Research Programme since 1998. Among children, 1–2 mLs of blood were obtained, where possible, for culture under aseptic conditions and were inoculated into a single aerobic bottle (BacT/Alert, bioMérieux, Marcy-L’Etoile, France). All lab data over the study period were collected for children ≤5 years, including young infants <60 days. Further clinical data was unavailable. Where possible, all duplicates were removed.

The automated BacT/Alert system (bioMérieux, France) has been used to incubate samples since 2000. Before then, manual cultures were used, as previously described [17], with organism identification using Analytical Profile Index (Biomérieux). Staphylococci were identified by slide coagulase, beta-hemolytic Streptococci by Lancefield antigen testing, and Salmonellae by serotyping, according to the White-Kauffmann-Le Minor scheme. *Haemophilus influenzae* were typed using type B antisera. *Aerococcus* spp., *Alcaligenes* spp., alpha-hemolytic streptococci (other than *S. pneumoniae*), *Bacillus* spp., Corynebacteria, *Micrococcus* spp., coagulase-negative staphylococci, unidentified gram-positive rods, and the *Rhizobium* spp. that form part of the normal skin or oral flora were considered contaminants.

In Malawi, first-line antimicrobial regimens for the treatment of pediatric and neonatal BSI include ampicillin/penicillin with gentamicin, or ceftriaxone. Antimicrobial susceptibility was determined by the disc diffusion method (Oxoid, United Kingdom), following the current version of the British Society of Antimicrobial Chemotherapy’s guidelines (http://www.bsac.org.uk). Intermediate susceptibility (with the exception of *S. pneumoniae*) was regarded as resistant. Methicillin resistance in *S. aureus* was inferred by resistance to cefoxitin, which replaced oxacillin resistance testing in 2010. For *S. pneumoniae*, reduced susceptibility to penicillin was detected by oxacillin disc, with resistance defined as a minimum inhibitory concentration >2 µg/mL. Formal minimum inhibitory concentration testing was not done. Since 2007, gram-negative isolates have been screened for their ESBL-producing status using a cefpodoxime disc. Prior to this, the ESBL-producing status was inferred based on any resistance to ceftriaxone.

### Statistical Analysis

We estimated minimum annual incidence rates, expressed as incidences per 1000 age-specific person years, by dividing the number of bacteremia cases per year by the mid-year population and multiplying by 1000. We modeled the observed annual case frequencies and then estimated incidences by dividing the predicted case frequencies by the mid-year populations. Age-stratified population estimates for urban Blantyre for the years 1998–2017 were obtained from the 1998 and 2008 National Population Projections by the National Statistical Office (http://www.nsomalawi.mw). We used yearly values for children ≤5 years, but when there were low numbers of cases, including for young infants ≤60 days, we combined data into 5-year periods to enable comparisons. We followed the WHO definition of young infants as ≤60 days [4] and defined early-onset neonatal BSI as <7 days and late-onset neonatal BSI as 7–90 days. We used negative, binomial regression models to test for linear trends in incidence rates and the Cochran-Armitage test to detect trends in AMR rates. All statistical analyses were performed using R Statistical Package version 3.3.2 for MacOS (R Core Team, http://www.r-project.org).

## RESULTS

Between 1998–2017, a total of 89643 blood cultures from pediatric patients were identified. Of these, 10621 pathogenic bacteria were identified from children ≤5 years, including 2898 from young infants ≤60 days.

### Incidence Rates

**Figure 1.**
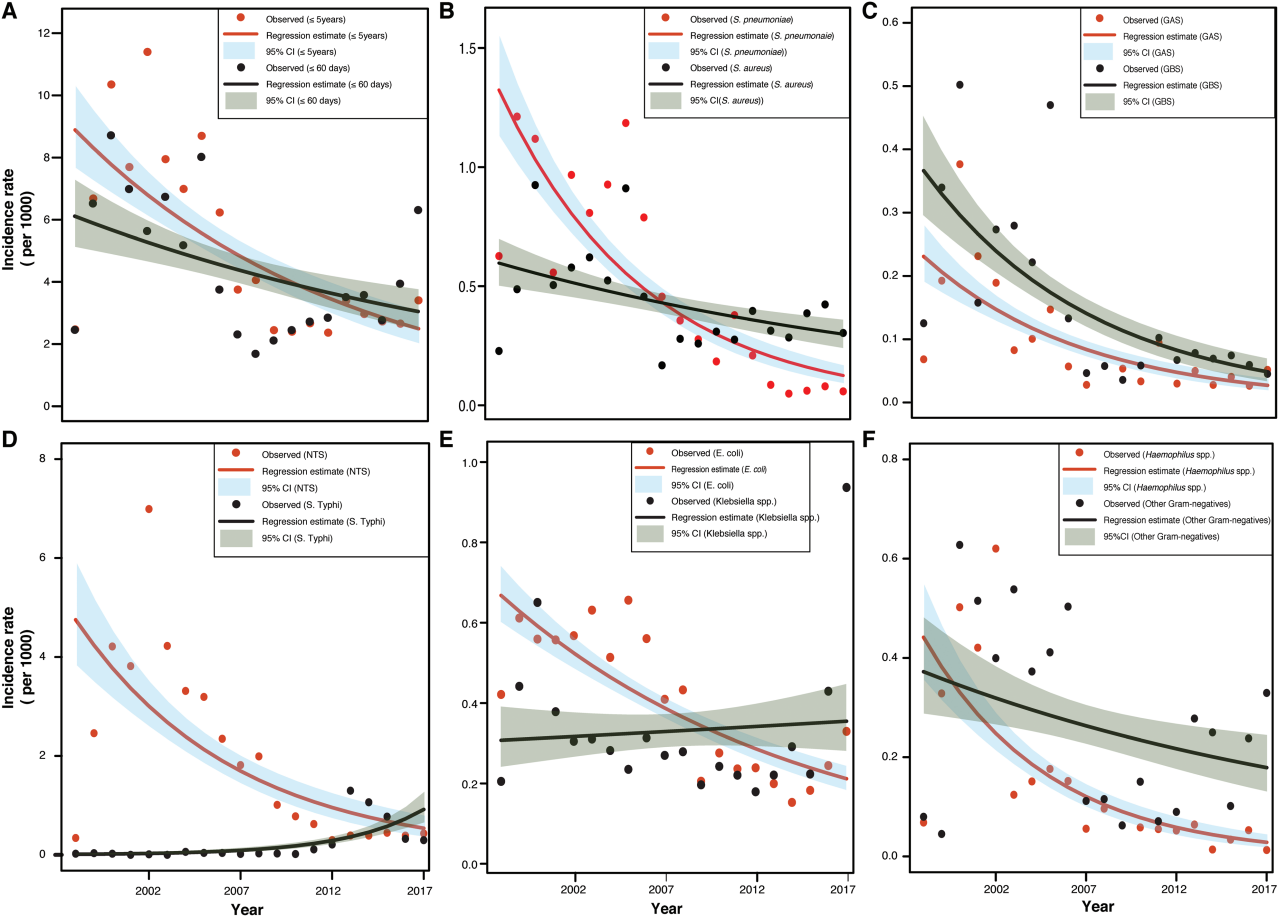
Negative binomial regression model–estimated annual incidence rate per 1000 person-years for children ≤5 years, for: (*A*) all pathogenic organisms among those ≤5 years and ≤60 days; (*B*) *S. pneumoniae* and *S. aureus*; (*C*) GAS and GBS; (*D*) *S.* Typhi and NTS; (*E*) *E. coli* and *Klebsiella* spp.; and (*F*) *Haemophilus* spp. and other Gram-negatives. Scales have been adjusted for each organism. Abbreviations: CI, confidence interval; GAS, Group A Strep; GBS, Group B Strep; NTS, non-typhoidal *Salmonella*.

Minimum incidence rates of pediatric BSI decreased significantly over 2 decades (Figure 1), falling from a peak of 11.4 per 1000 persons in 2002 for children ≤5 years to 3.4 per 1000 persons in 2017 (overall decreasing trend, *P* < .001). For young infants, minimum incidence rates decreased from 8.7 per 1000 persons in 2000 to 1.7 per 1000 persons in 2008 (*P* = .05). However, since 2008, minimum incidence rates for young infants have been rising, with a steep rise from 2.8 per 1000 persons in 2015 to 6.3 per 1000 persons in 2017. For most pathogens, there is an overall decreasing trend in minimum incidence rates (Figure 1) over these 2 decades, with the exception of *Salmonella* Typhi and *Klebsiella* spp. For *S.* Typhi, minimum incidence rates for children ≤5 years increased from a low of 0 per 1000 persons in 2003 to a peak of 1.3 per 1000 persons in 2013, and then declined to 0.3 per 1000 persons in 2017 (overall increasing trend, *P* = .0016); for *Klebsiella* spp., minimum incidence rates went from a low of 0.2 per 1000 persons in 2012 to 0.9 per 1000 persons in 2017 (*P* = .727). However, when the outlier values for 2016 and 2017 were excluded, there was a declining trend for *Klebsiella* spp. sepsis between 1998 and 2015 (*P* = .009).

### Pathogen Etiology

During the first period (1998–2002; Table 1), NTS accounted for 41.4% (1644/3964) of pathogenic isolates, followed by *S. pneumoniae* at 10.2% (405/3964) and *S. aureus* and *E. coli* at 6.2% each (247/3964). In the last period (2013–2017), the most common causes of pediatric BSI were *Salmonella* Typhi (544/2614, 20.8%), *Klebsiella* spp. (316/2614, 12.1%, with *K. pneumoniae* comprising 93.7% [295] of these), NTS (303/2614, 11.6%), and *Staphylococcus aureus* (253/2614, 9.7%). *S. pneumoniae* accounted for 1.9% (49/2614) of isolates in the last period. For young-infant BSI in the first period (Supplementary Table 2), the most common causes were NTS (133/835, 15.9%), *S. aureus* (118/835, 14.1%), and Group B Strep (109/835, 13.1%). In the last period, the most common causes were *Klebsiella* spp. (230/800, 28.9%), followed by *S. aureus* (139/800, 17.4%), *Enterobacter* spp. (101/800, 12.6%), *Enterococcus* spp. (72/800, 9%), *E. coli* (57/800, 7.1%), and Group B Strep (43/800, 5.4%). Only 0.6% (5/800) of *S. pneumoniae* specimens were isolated from young infants in the last period, compared to 6.2% (52/835) in the first.

**Table 1.** Bloodstream Infections in Children ≤5 Years at Queen Elizabeth Central Hospital, by Isolate and Period

	Time Period							
	1998–2002		2003–2007		2008–2012		2013–2017	
	n	%	n	%	n	%	n	%
**Gram-positives**								
Group A Strep	96	2.4	42	1.1	32	1.7	29	1.1
Group B Strep	126	3.2	116	2.9	39	2.0	48	1.8
*Streptococcus pneumoniae*	405	10.2	423	10.7	166	8.6	49	1.9
*Staphylococcus aureus*	247	6.2	283	7.1	185	9.6	253	9.7
Other *Streptococcus* spp.	197	5.0	244	6.1	193	10.0	222	8.5
*E. faecalis*	26	0.7	63	1.6	38	2.0	30	1.1
*E. faecium*	0	0	0	0	10	0.5	69	2.6
All *Enterococcus* spp.	31	0.8	63	1.6	50	2.6	99	3.8
*Leuconostoc*	0	0	1	0.03	0	0	0	0
**Gram-negatives**								
*Acinetobacter baumanii*	0	0	9	0.2	17	0.9	58	2.2
All *Acinetobacter* spp.	86	2.2	101	2.5	35	1.8	59	2.3
*Citrobacter* spp.	37	0.9	33	0.8	6	0.3	7	0.3
*Enterobacter* spp.	57	1.4	133	3.4	33	1.7	132	5.0
*Escherichia coli*	247	6.2	282	7.1	163	8.5	165	6.3
*Haemophilus influenzae* type B	157	4.0	44	1.1	19	1.0	16	0.6
All *Haemophilus* spp.	178	4.5	67	1.7	38	2.0	26	1.0
*Klebsiella pneumoniae*	57	1.4	103	2.6	123	6.4	295	11.3
All *Klebsiella* spp.	179	4.5	144	3.6	132	6.9	316	12.1
*Neisseria meningitidis*	14	0.3	18	0.5	5	0.3	11	0.4
*N. gonorrhoea*	0	0	0	0	1	0.05	6	0.3
*Proteus* spp.	15	0.4	3	0.08	2	0.1	3	0.1
*Pseudomonas aeruginosa*	28	0.7	47	1.2	20	1.0	36	1.4
All *Pseudomonas* spp.	38	1.0	55	1.4	33	1.7	49	1.9
*Salmonella* typhi	8	0.2	16	0.4	50	2.6	544	20.8
NTS	1644	41.4	1505	37.9	532	27.7	303	11.6
*Serratia* spp.	38	1.0	16	0.4	4	0.2	17	0.7
*Shigella* spp.	3	0.08	4	0.1	4	0.2	6	0.2
*Vibrio* spp.	0	0	0	0	2	0.1	0	0
*Yersinia* spp.	2	0.05	0	0	2	0.1	0	0
Other Gram-negatives^a^	301	7.6	412	10.4	203	10.6	253	9.7
**Fungus**								
*Candida*	5	0.1	1	0.03	4	0.2	7	0.3
*Cryptococcus*	0	0	0	0	6	0.3	4	0.2
Yeast species	0	0	0	0	0	0	3	0.1
All pathogens^b^	3964	100	3970	100	1923	100	2614	100

Abbreviation: NTS, non-typhoidal Salmonella.

^a^Includes *Aeromonas* spp., *Agrobacter* spp., *Burkholderia* spp., *Cronobacter* spp., *Edwardsiella* spp., Flavobacteria, Gram-negative rods, *Hafnia* spp., *Histophilus* spp., *Kluyvera* spp., *Moraxella* spp., *Morganella* spp., *Pantoea* spp., *Pasteurella* spp., *Raoultella* spp., *Sphingomonas* spp., *Stenotrophomonas* spp., and *Xanthomonas* spp.

^b^Excludes contaminants, including *Aerococcus* spp., alpha-hemolytic streptococci, *Alcaligenes* spp., *Bacillus* spp., *Clostridium* spp., coagulase-negative staphylococci, Corynebacteria, Diphtheroids, Gram-positive rods, *Rhizobium* spp., *Micrococcus* spp., and skin flora.

### Antimicrobial Resistance Profiles

**Figure 2.**
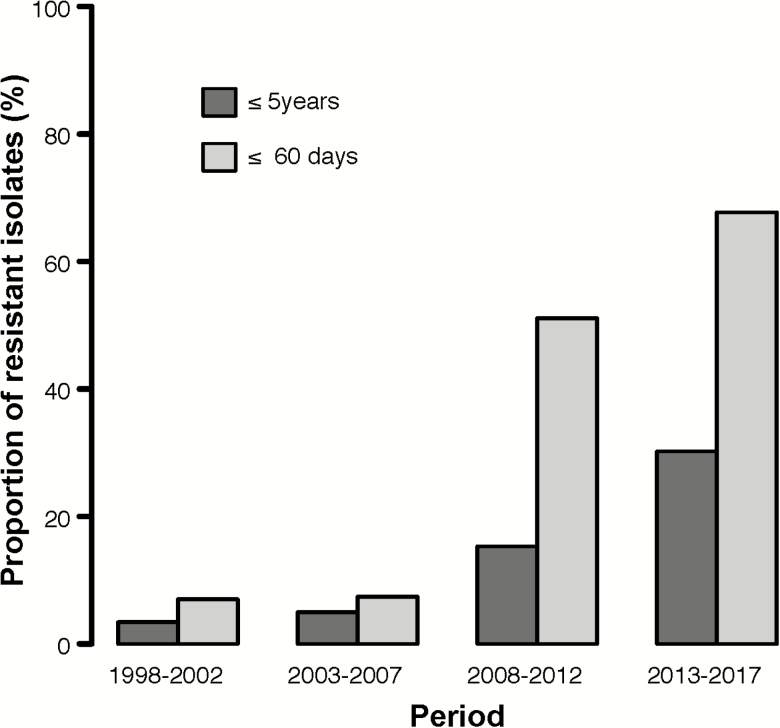
Proportion of culture-confirmed bloodstream pathogens resistant to empiric first-line antimicrobials by period, for children ≤5 years and ≤60 days. First-line antimicrobials in Malawi are ampicillin/penicillin with gentamicin, or ceftriaxone.

For Gram-positive pathogens, resistance to empiric, first-line antimicrobials—ampicillin/penicillin with gentamicin, or ceftriaxone—was 21.1% (605/2863) and 6.2% (74/1199) of isolates, respectively. For Gram-negative pathogens, the proportion of culture-confirmed BSI among children ≤5 years that were resistant to all first-line antimicrobials had an overall increasing trend over time, from 3.4% (8/235) in the first period to 30.2% (449/1487) in the last period (*P* < .001; Figure 2). For young infants, the overall proportion of Gram-negative bacteria resistant to all first-line antimicrobials increased from 7.0% (3/43) in the first period to 67.7% (315/465) in the last (*P* < .001).

For Gram-positive pathogens, penicillin resistance (including intermediate susceptibility) in *S. pneumoniae* did not increase significantly during the study period (*P* = .210; Table 2). Methicillin-resistant *Staphylococcus aureus* isolates increased from 0% (0/247) to 2.7% (6/224), which was not significant (*P* = .09). *Enterococcus* spp. resistance to ampicillin increased from 11.1% (1/9) in the first period to 69.7% (69/99) in the last (*P* < .001), which could be attributed to the 7-fold increase in *E. faecium* spp. isolated, concurrent with a relative decrease in *E. faecalis* over the same period.

**Table 2.** Antimicrobial Resistance Profiles of Selected Bloodstream Pathogens for Children ≤5 Years, by Period

Pathogen	Time Period	Antimicrobial Resistance													
		Ampicillin		Penicillin		Ceftriaxone		Chloramphenicol		Gentamicin		Co-trimoxazole		Ciprofloxacin	
		n	%	n	%	n	%	n	%	n	%	n	%	n	%
*S. pneumoniae*	1998–2002	NT		91/ 374	24.3	0^a^/ 50	0	56/ 374	15.0	NT		314/ 364	82.3	NT	
	2003–2007	NT		73/ 420	17.4	0^a^/ 421	0	72/ 418	17.2	NT		387/ 411	94.2	NT	
	2008–2012	NT		29/ 164	17.7	0^a^/ 166	0	21/ 164	17.7	NT		153/ 166	92.3	NT	
	2013–2017	NT		15/ 49	30.6	0^a^/ 48	0	15/ 49	30.6	NT		46/49	93.9	NT	
*S. aureus* ^b^	1998–2002	NT		227/ 245	92.7	NT		97/ 241	40.2	27^c^/ 236	11.4	127/ 244	52.0	NT	
	2003–2007	NT		245/ 262	93.5	NT		85/ 269	31.6	28^c^/ 222	12.6	137/ 265	51.7	NT	
	2008–2012	NT		47/ 57	82.5	NT		29/ 181	16.0	20^c^/ 160	12.5	78/ 180	43.3	NT	
	2013–2017	NT		NT		NT		12/ 253	4.7	20^c^/ 224	8.9	96/ 253	37.9	NT	
*Enterococcus* spp.	1998–2002	1/ 9	11.1	16/ 27	59.3	NT		14/ 30	46.7	20/ 22	90.9	17/ 31	54.8	NT	
	2003–2007	5/ 44	11.4	28/ 32	87.5	NT		38/ 62	61.3	8/ 8	100	33/ 61	54.1	NT	
	2008–2012	17/ 38	44.7	2/ 2	100	NT		33/ 47	70.2	2/ 3	66.7	40/ 44	90.9	NT	
	2013–2017	69/ 99	69.7	NT		NT		74/ 98	75.5	1/ 1	100	73/ 98	74.5	NT	
*E. coli*	1998–2002	222/ 246	90.2	NT		4/ 36	11.1	172/ 243	70.8	48/ 244	19.7	213/ 242	88.0	0/ 113	0
	2003–2007	237/ 272	87.1	NT		14/ 237	5.9	178/ 277	64.3	74/ 274	27.0	244/ 271	90.0	9/ 275	3.3
	2008–2012	147/ 162	90.7	NT		24/ 154	15.6	99/ 162	61.1	57/ 161	35.4	150/ 162	92.6	23/ 161	14.3
	2013–2017	147/ 163	90.2	NT		47/ 165	28.5	72/ 165	43.6	54/ 165	32.7	160/ 165	97.0	43/ 165	26.0
*Klebsiella* spp.	1998–2002	178/ 178	100	NT		4/ 19	21.1	139/ 177	78.5	68/ 178	38.2	149/ 176	84.7	0/ 73	0
	2003–2007	136/ 140	97.1	NT		18/ 123	14.6	98/ 139	70.5	72/ 144	50	109/ 140	77.9	11/ 142	7.7
	2008–2012	130/ 131	99.2	NT		90/ 118	76.3	85/ 129	65.9	105/ 130	80.8	116/ 131	88.5	26/ 131	19.8
	2013–2017	316/ 316	100	NT		286/ 316	90.5	167/ 315	53.0	282/ 311	90.7	299/ 315	94.9	109/ 316	34.5
*Enterobacter* spp.	1998–2002	48/ 53	90.6	NT			100^d^	34/ 57	59.6	19/ 55	34.5	36/ 56	64.3	1/ 40	2.5
	2003–2007	95/ 122	77.9	NT			100^d^	70/ 131	53.4	33/ 114	28.9	79/ 131	60.3	7/ 119	5.9
	2008–2012	29/ 31	93.5	NT			100^d^	22/ 32	68.8	21/ 32	65.6	27/ 32	84.4	18/ 32	56.3
	2013–2017	126/132	95.5	NT			100^d^	120/ 132	90.9	101/ 132	76.5	115/ 132	87.1	87/ 132	65.9
*Acinetobacter* spp.	1998–2002	55/ 83	66.3	NT		3/ 12	25	54/ 84	64.3	30/ 84	35.7	57/ 85	67.1	2/ 38	5.3
	2003–2007	70/ 95	73.7	NT		45/ 95	47.4	82/ 100	82	43/ 101	42.6	85/ 98	86.7	26/ 101	25.7
	2008–2012	25/ 32	78.1	NT		21/ 31	67.7	30/ 34	88.2	16/ 33	48.5	27/ 35	77.1	9/ 33	27.3
	2013–2017	45/ 59	76.3	NT		57/ 59	96.6	52 /59	88.1	26/ 59	44.1	40/ 59	67.8	27/ 59	45.8
*Pseudomonas* spp.	1998–2002	NT^d^		NT		NT		NT^e^		6/ 38	15.8	NT^d^		0/ 24	0
	2003–2007	NT^d^		NT		NT		NT^e^		20/ 55	36.4	NT^d^		2/ 55	3.6
	2008–2012	NT^d^		NT		NT		NT^e^		8/ 31	25.8	NT^d^		5/ 31	16.1
	2013–2017	NT^d^		NT		NT		NT^e^		12/ 49	24.5	NT^d^		8/ 49	16.3
*Salmonella* Typhi	1998–2002	4/ 7	57.1	NT		0/ 2	0	3/ 7	42.9		100^f^	4/ 7	57.1	0/ 1	0
	2003–2007	2/ 16	12.5	NT		0/ 16	0	0/ 16	0		100^f^	0/ 16	0	0/ 15	0
	2008–2012	37/ 50	74	NT		0/ 50	0	37/ 50	74		100^f^	35/ 50	70	1/ 50	2
	2013–2017	532/ 544	97.8	NT		0/ 498	0	526/ 544	96.7		100^f^	533/ 544	98.0	0/ 544	0
NTS	1998–2002	1481/ 1603	92.4	NT		0/ 41	0	904/ 1609	56.1		100^f^	1430/ 1604	89.2	1/ 1070	0.1
	2003–2007	1382/ 1488	92.9	NT		3/ 1142	0.3	1297/ 1497	86.6		100^f^	1317/ 1437	91.6	9/ 1495	0.6
	2008–2012	459/ 527	87.1	NT		2/ 495	0.4	415/ 526	78.9		100^f^	456/ 528	86.4	0/ 524	0
	2013–2017	241/ 303	79.5	NT		3/ 302	1.0	177/ 303	58.4		100^f^	238/ 303	78.5	0/ 303	0
Other Entero- bacteriaceae^g^	1998–2002	94/ 102	92.2	NT		1/ 2	50	82/ 104	78.8	26/ 105	24.8	82/ 105	78.1	1/ 70	1.4
	2003–2007	59/ 69	85.5	NT		6/ 55	10.9	43/ 69	42.0	16/ 70	22.9	37/ 62	59.7	8/ 69	11.6
	2008–2012	16/ 24	66.7	NT		4/ 20	20	8/ 24	33.3	6/ 21	28.6	15/ 24	62.5	5/ 23	21.7
	2013–2017	33/ 42	78.6	NT		18/ 42	42.9	19/ 42	45.2	13/ 41	31.7	29/ 41	70.7	12/ 42	28.6
Other Gram- negatives^h^	1998–2002	105/ 145	72.4	0/ 12	0	4/ 123	3.3	75/ 161	46.6	11/ 159	6.9	141/ 158	89.2	1/ 12	8.3
	2003–2007	54/ 93	58.1	4/ 23	17.4	4/ 121	0.3	64/ 122	52.5	21/ 118	17.8	85/ 109	78.0	5/ 77	6.5
	2008–2012	52/ 76	68.4	5/ 7	71.4	18/ 109	16.5	42/ 84	50	31/ 108	28.7	64/ 86	74.4	17/ 70	24.3
	2013–2017	44/ 52	84.6	4/ 9	44.4	25/ 75	33.3	31/ 63	49.2	23/ 57	40.4	38/ 50	76	10/ 50	20

Abbreviations: NT, not tested; NTS, nontyphoidal Salmonella.

^a^Pneumococcal isolates initially reported as ceftriaxone-resistant were re-tested and found to be susceptible. Based on these, isolates not available for re-testing (9) were considered susceptible.

^b^Methicillin-resistant *Staphylococcus aureus* isolates for these periods were: 1998–2002, 0 (0%); 2003–2007, 12 (0.3%); 2008–2012, 20 (1.0%); and 2013–2017, 35 (1.3%).

^c^Note that gentamicin should not be used alone for *S. aureus,* even if susceptible.

^d^All Enterobacter isolates have been reported resistant to ceftriaxone, in line with British Society of Antimicrobial Chemotherapy (BSAC) guidance.

^e^All Pseudomonas isolates have been reported not tested, as these pathogens are intrinsically resistant to ampicillin, chloramphenicol, and co-trimoxazole.

^f^All Salmonella isolates have been reported resistant to gentamicin, in line with BSAC guidance.

^g^Includes *Citrobacter* spp., Coliforms, *Cronobacter* spp., *Escherichia* spp., *Hafnia* spp., *Kluyvera* spp., *Morganella* spp., *Pantoea* spp., *Proteus* spp., *Raoultella* spp., *Serratia* spp., *Shigella* spp., and *Yersinia* spp.

^h^Includes *Aeromonas* spp., *Burkholderia* spp., *Edwardsiella* spp., Flavobacteria, Gram-negative rods, *Haemophilus* spp., *Histophilus* spp., *Moraxella* spp., *Neisseria* spp., *Pasteurella* spp., *Sphingomonas* spp., *Stenotrophomonas* spp., *Vibrio* spp., and *Xanthomonas* spp.

**Figure 3.**
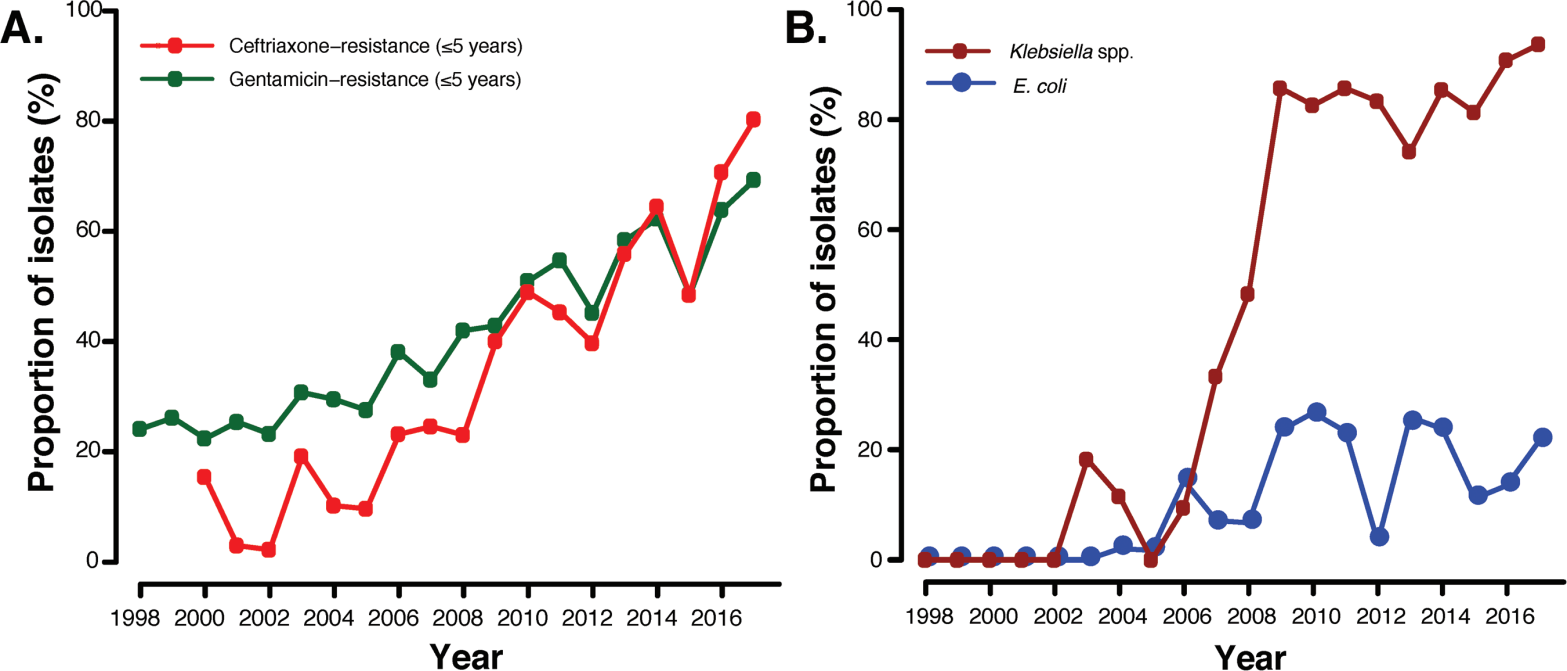
Proportion of non-Salmonella, Gram-negative isolates with: (*A*) Gentamicin- or ceftriaxone-resistance, for children ≤5 years; or (*B*) *E. coli* and *Klebsiella* spp. resistance to empiric first-line antimicrobials, for children ≤5 years. First-line antimicrobials in Malawi are ampicillin/penicillin with gentamicin, or ceftriaxone.

For Gram-negative pathogens, excluding Salmonellae, an increase in resistance to ceftriaxone and gentamicin to over 60% during the study period was documented (Figure 3). *E. coli’s* resistance to ceftriaxone increased from 11.1% (4/36) in the first period to 28.5% (47/165) in the last (*P* < .001), while its resistance to gentamicin increased from 19.7% (48/244) in the first period to 32.7% (54/165) in the last (*P* < .001), and its resistance to ampicillin remained unchanged and had a mean annual rate of 89.7% throughout the study period (*P* = .466). For *Klebsiella* spp., ceftriaxone resistance rose from 21.1% (4/19) in the first period to 90.5% (286/316) in the last (overall increasing trend, *P* < .001), whereas gentamicin resistance rose from 38.2% (68/178) in the first period to 90.7% (282/311) in the last (overall increasing trend, *P* < .001). Resistance to ciprofloxacin for both pathogens was also observed to steadily increase during the entire 2 decades, from 0% (0/113) in the first period to 26.0% (43/165) in the last (*P* < .001) in *E. coli* and from 0% (0/73) to 34.5% (109/316) for *Klebsiella* spp (*P* < .001). Resistance to all empiric, first-line antimicrobials for *E. coli* was over 20% (Figure 3) and there was an overall increasing trend in the proportion of *Klebsiella* spp. resistant to all first-line antimicrobial regimens over time, from 5.9% (1/17) to 93.7% (133/142; *P* < .001). Both *S*. Typhi and NTS had infrequent resistances to ceftriaxone (0/566 and 8/1980) and ciprofloxacin (1/610 and 10/3392) throughout all periods. We have not detected ESBL NTS BSI in children [18]. A similar trend in AMR profiles was also noted for young infants (Supplementary Table 3).

## DISCUSSION

Over the past 2 decades, overall, the incidence of BSI in hospitalized children at QECH, the largest teaching hospital in Malawi, has decreased. This includes NTS, for which no vaccine was available during the study period, and *H. influenzae* type B and *S. pneumoniae*, for which vaccination programs were introduced during the study period (in 2002 and 2011, respectively). However, in the last 10 years, the incidence of young infant BSI caused by *S.* Typhi and *Klebsiella* spp. in those under 5 has risen considerably. We have previously reported 3 epidemics of *Salmonella*, which appear to have arisen through the acquisition of virulence and AMR determinants in the context of a susceptible population [19–22]. Gram-positive pathogens are still largely susceptible to first-line antimicrobials, with the exception of *Enterococcus* spp. and *E. faecium*, which, as an emerging pathogen, needs ongoing surveillance. For Gram-negative BSI among children ≤5 years, the proportion of bacteria that are resistant to empiric, first-line antimicrobials is high, rising, and most marked among young infants. These findings reveal the growing problem of AMR in our setting and emphasize the need for robust antimicrobial stewardship programs and ongoing surveillance, even when BSI overall appears to be declining.

The rise in the number of Gram-negative pathogens resistant to first-line antimicrobials, specifically *Klebsiella* spp., contrasts with earlier data from our institution, where isolates had high susceptibility (78%) [23]. One reason for the rise in AMR could be attributed to a marked increase in the use of broad-spectrum antimicrobials in recent years [24], including among neonates. *Klebsiella* spp. isolates recovered within the first 3 days of birth accounted for 28.4% (84/295) of all pediatric *Klebsiella* spp. specimens in the last period, suggesting high rates of vertical transmission. The reasons for increases in rates of AMR *Klebsiella* spp. are not certain, since there have been no changes in practices in the neonatal unit that we can identify. The increase may have been amplified by an increased transmission capacity, resulting in outbreaks in neonatal units [25, 26]. The high ESBL carriage in children <5 years [27] may have also contributed to horizontal transmission. Community-based surveillance of both the disease and carriage is ongoing [28] to explore whether increasing rates of AMR may be related to the strengthening of primary healthcare systems following the model of IMCI and earlier treatment with antimicrobials, and may thereby be limited to the hospital setting.

Alternative antimicrobials for Gram-negative pathogens in these settings could include the use of amikacin, where resistance rates are much lower than gentamicin (6.3% compared to 66.0%, respectively); or ciprofloxacin, where resistance rates are already rising, although, when used in children, this has to be weighed against the side effects of tendinopathy. The inclusion of meropenem on the Malawi national formulary in 2015 and its availability in hospitals may be beneficial in the short term, but increased use is likely to drive further AMR, similar to the increase in ESBL organisms seen following ceftriaxone use. This would be in a setting where there are limited diagnostic facilities and where antimicrobial stewardship initiatives are beset by scarce resources.

Limitations to this study include that these are urban, tertiary, referral, hospital-based data from a single site, and not national surveillance. However, such large-scale, long-term, routine surveillance data is rarely available in this setting at a national level. Hospital-based surveillance could have impacted the rates of AMR, as susceptible BSI may have responded to empiric oral antibiotics in the community, and only the sickest children present to the hospital. However, given the likely clinical severity of BSI, except for in children with typhoid fever, and the absence of other pediatric inpatient facilities, this bias is unlikely to be substantial. We could not reliably differentiate community- from hospital-acquired BSI, which could be particularly relevant to young-infant BSI, where some neonates have prolonged stays and have follow-up blood cultures obtained prior to changes in antimicrobial regimens for presumed sepsis. Nosocomial BSI may have contributed to our relatively high recovery rate of 11.9%, but many of the prominent pathogens are uncommonly associated with nosocomial transmission. As blood cultures are not routinely repeated if the bacteria that are considered contaminants are isolated, we may have underestimated the number of true pathogens; however, typical risk factors, such as indwelling vascular devices or febrile neutropenia, are extremely rare in this population and prematurity would not have accounted for a substantial proportion. ESBL screening was not introduced until 2003; therefore, ESBL-producing pathogens may have been circulating yet undetected before then. Changes made to breakpoints in subsequent British Society of Antimicrobial Chemotherapy versions would have led to a wider use of “intermediate” categories that, in our study, may have increased the number of Gram-negative pathogens regarded as resistant. In all pediatric BSI surveillance studies, the available blood volumes for culture remain a challenge and, therefore, underestimates of pathogens such as *S. pneumoniae* and *S.* Typhi are possible, particularly in infants. Comprehensive clinical data was not available to evaluate the contribution of AMR to mortality; however, we have previously reported the mortality associated with specific pathogens [29–32].

Our results raise significant concerns about the growing issue of AMR, and highlight the urgent need to review empiric, antimicrobial regimens; implement and enforce infection control practices; and undertake pragmatic trials of antimicrobial stewardship. However, in many LMICs, without local data, it remains challenging to adapt the WHO IMCI guidelines to reflect local needs [5]. While awaiting both the impact of international initiatives to expand access to high-quality laboratory surveillance [33, 34] and the development of rapid diagnostics [35, 36], there should be a considerable emphasis on expanded access to effective antimicrobials in the context of locally-relevant antimicrobial stewardship programs. These should include the education of community- and hospital-based healthcare providers on prescribing practices, the epidemiology of infections, when not to prescribe antibiotics, the development of and adherence to guidelines on de-escalation and cessation of antimicrobial therapy, and infection control practices. The development of pragmatic clinical trials to evaluate new antimicrobial strategies in young children should be a priority.

## Supplementary Data

Supplementary materials are available at *Clinical Infectious Diseases* online. Consisting of data provided by the authors to benefit the reader, the posted materials are not copyedited and are the sole responsibility of the authors, so questions or comments should be addressed to the corresponding author.

ciy834_suppl_Supplementary_Figure_1

ciy834_suppl_Supplementary_Table_1

ciy834_suppl_Supplementary_Table_2

ciy834_suppl_Supplementary_Table_3

ciy834_suppl_Supplementary_Legends

## Notes

***Acknowledgments.*** The authors thank the clinical and support staff at Queen Elizabeth Central Hospital. They thank Todd Swarthout and Jacquline Chinkwita for assistance with antimicrobial susceptibility re-testing of pneumococcal isolates. They thank Franziska Olgemoeller for collating pediatric antibiogram data. They thank Clemens Masesa and Moses Kamzati for extraction of blood culture data.

***Financial support.*** This work was supported by the Wellcome Trust; National Institutes of Health through the H3ABionet Project (grant number U41HG006941 to D. E.); and the Southern Africa Consortium for Research Excellence.

***Potential conflicts of interest.*** N. French reports grants from GlaxoSmithKline, outside the submitted work. P. I. reports grants from the Bill and Melinda Gates Foundation, outside the submitted work. All other authors report no potential conflicts.
