# Supplementary figures and images for: Emerging Resistance to Empiric Antimicrobial Regimens for Pediatric Bloodstream Infections in Malawi (1998–2017)
Source: Clin Infect Dis. 2018 Oct 1;69(1):61–8. doi: 10.1093/cid/ciy834 (PMC6579959; doi:10.1093/cid/ciy834)

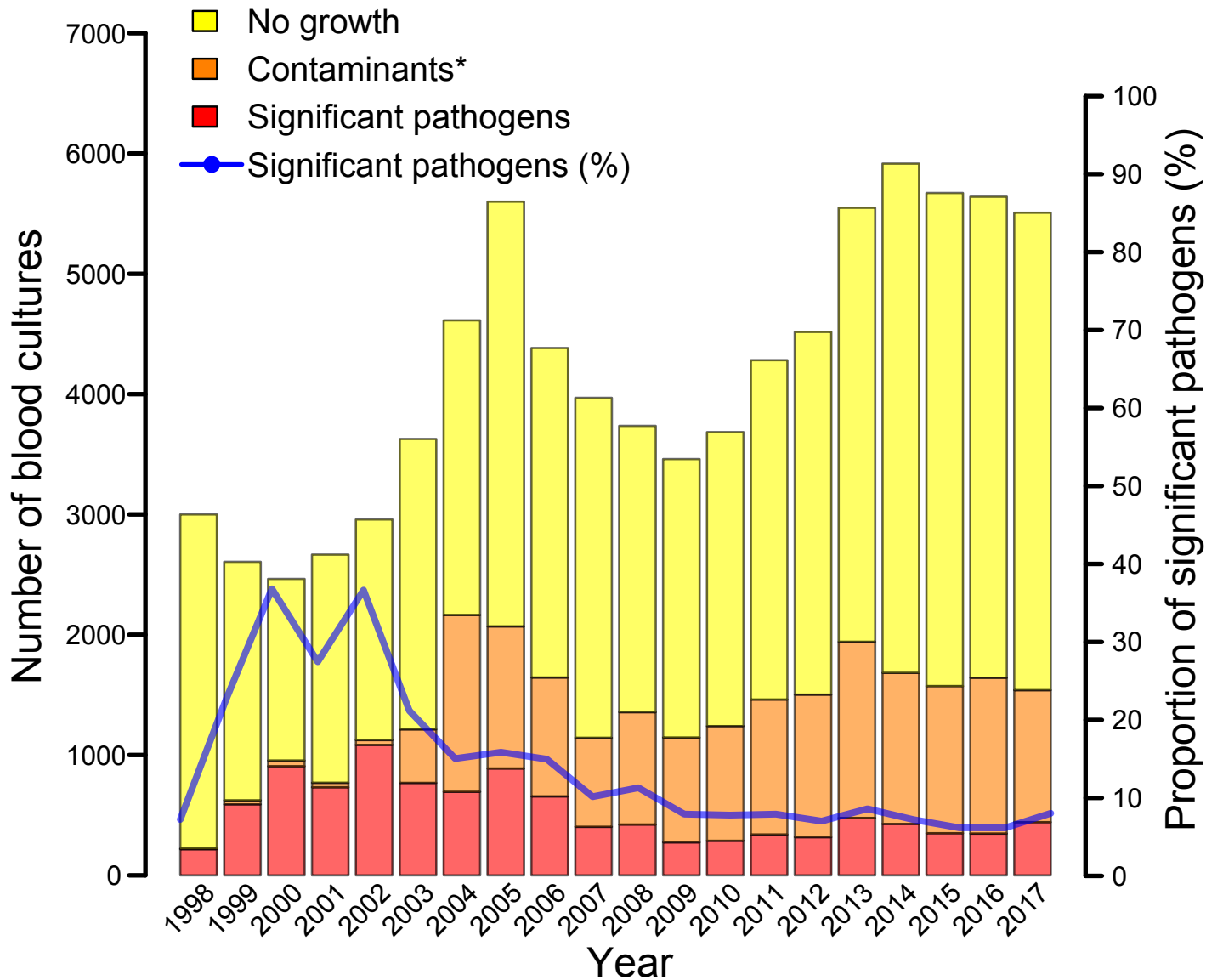

Supplement: ciy834_suppl_Supplementary_Figure_1 [file ciy834_suppl_supplementary_figure_1.pdf]
