# Supplementary material for: Emerging Resistance to Empiric Antimicrobial Regimens for Pediatric Bloodstream Infections in Malawi (1998–2017)
Source: Clin Infect Dis. 2018 Oct 1;69(1):61–8. doi: 10.1093/cid/ciy834 (PMC6579959; doi:10.1093/cid/ciy834)
Supplement: ciy834_suppl_Supplementary_Table_1 [file ciy834_suppl_supplementary_table_1.doc]

**Supplementary Table 1.** Admission statistics in Queen Elizabeth Central Hospital, by ward and year

| Year | Total pediatric hospital admissions | Chatinkha  all-cause admissions  (% of total admissions) |
| --- | --- | --- |
| 2002 | 17,051 | NA |
| 2003 | 19,819 | NA |
| 2004 | 18,618 | NA |
| 2005 | 22,326 | 1,501^a^ (6.7) |
| 2006 | 24,074 | 2,771 (11.5) |
| 2007 | 28,214 | 3,010 (10.7) |
| 2008 | 27,481 | 3,317 (12.1) |
| 2009 | 27,145 | 3,252 (12.0) |
| 2010 | 27,968 | 3,738 (13.4) |
| 2011 | 30,888 | 3,914 (12.7) |
| 2012 | 28,323 | 3,887 (13.7) |
| 2013 | 26,066 | 3,541 (13.6) |
| 2014 | 22,831 | 3,914 (17.1) |
| 2015 | 24,759 | 4,312 (17.4) |
| 2016 | 22,501 | 4,220 (18.8) |
| 2017 | 10,611^b^ | 3,564^c^ (18.5)^d^ |

NA, not available

^a^Data from July-Dec 2005 only

^b^Data from Jan-Jun 2017 only

^c^Data from Jan-Nov 2017 only

^d^Calculated using data from Jan-Jun 2017 (1,965 admissions) only
