# Supplementary material for: Emerging Resistance to Empiric Antimicrobial Regimens for Pediatric Bloodstream Infections in Malawi (1998–2017)
Source: Clin Infect Dis. 2018 Oct 1;69(1):61–8. doi: 10.1093/cid/ciy834 (PMC6579959; doi:10.1093/cid/ciy834)
Supplement: ciy834_suppl_Supplementary_Table_2 [file ciy834_suppl_supplementary_table_2.docx]

**Supplementary Table 2.** Bloodstream infections in children ≤60 days at Queen Elizabeth Central Hospital, by isolate and year

|  | Time period | | | | | | | |
| --- | --- | --- | --- | --- | --- | --- | --- | --- |
|  | 1998-2002 | | 2003-2007 | | 2008-2012 | | 2013-2017 | |
|  | N | % | N | % | N | % | N | % |
| **Gram-positives** |  |  |  |  |  |  |  |  |
| Group A Strep | 66 | 7.9 | 23 | 2.8 | 17 | 4.0 | 9 | 1.1 |
| Group B Strep | 109 | 13.1 | 108 | 13.3 | 37 | 8.7 | 43 | 5.4 |
| *Streptococcus pneumoniae* | 52 | 6.2 | 57 | 7.0 | 23 | 5.4 | 5 | 0.6 |
| *Staphylococcus aureus* | 118 | 14.1 | 130 | 16.0 | 82 | 19.4 | 139 | 17.4 |
| Other *Streptococcus* spp. | 24 | 2.9 | 17 | 2.1 | 11 | 2.6 | 25 | 3.1 |
| *Enterococcus* spp. | 24 | 2.9 | 43 | 5.3 | 30 | 7.1 | 72 | 9 |
| **Gram-negatives** |  |  |  |  |  |  |  |  |
| *Acinetobacter* spp. | 32 | 3.8 | 27 | 3.3 | 17 | 4.0 | 35 | 4.4 |
| *Citrobacter* spp. | 14 | 1.7 | 22 | 2.7 | 2 | 0.5 | 4 | 0.5 |
| *Enterobacter* spp. | 29 | 3.5 | 69 | 8.5 | 17 | 4.0 | 101 | 12.6 |
| *Escherichia coli* | 74 | 8.9 | 95 | 11.7 | 47 | 11.1 | 57 | 7.1 |
| *Haemophilus* spp. | 12 | 1.4 | 6 | 0.7 | 1 | 0.2 | 3 | 0.4 |
| *Klebsiella* spp. | 98 | 11.7 | 62 | 7.6 | 77 | 18.2 | 230 | 28.9 |
| *Neisseria* spp. | 1 | 0.1 | 1 | 0.1 | 2 | 0.5 | 1 | 0.1 |
| *Proteus* spp. | 8 | 1.0 | 3 | 0.4 | 0 | 0 | 0 | 0 |
| *Pseudomonas* spp. | 22 | 2.6 | 25 | 3.1 | 16 | 3.8 | 26 | 3.3 |
| *Salmonella* Typhi | 0 | 0 | 0 | 0 | 2 | 0.5 | 7 | 0.9 |
| NTS | 133 | 15.9 | 101 | 12.5 | 18 | 4.3 | 9 | 1.1 |
| *Serratia* spp. | 10 | 1.2 | 6 | 0.7 | 1 | 0.2 | 8 | 1 |
| Other *Enterobacteriaceae^a^* | 3 | 0.4 | 1 | 0.1 | 1 | 0.2 | 6 | 0.8 |
| Other Gram-negatives^b^ | 6 | 0.7 | 14 | 1.7 | 17 | 4.0 | 17 | 2.1 |
| **Fungus** |  |  |  |  |  |  |  |  |
| *Candida* | 0 | 0 | 1 | 0.1 | 2 | 0.5 | 2 | 0.3 |
| *Cryptococcus* | 0 | 0 | 0 | 0 | 3 | 0.7 | 1 | 0.1 |
| All pathogens^c^ | 835 | 100 | 811 | 100 | 423 | 100 | 800 | 100 |

NTS, nontyphoidal Salmonella

^a^Includes *Kluyvera* spp., *Morganella* spp., *Pantoea* spp., *Raoultella* spp., *Shigella* spp., *Yersinia* spp..

^b^Includes *Aeromonas* spp., *Burkholderia* spp., Coliforms, *Edwardsiella* spp., Gram negative rods, *Moraxella* spp., *Pasteurella* spp., *Stenotrophomonas* spp., *Xanthomonas* spp..

^c^Excludes contaminants, including *Aerococcus* spp., alpha-hemolytic streptococci, *Alcaligenes* spp., *Bacillus* spp., *Clostridium* spp., coagulase-negative staphylococci, Corynebacteria, Diphtheroids, Gram positive rods, *Micrococcus* spp., *Rhizobium* spp., skin flora.

**Supplementary Table 2B.** Bloodstream infections in children 7-90 days at Queen Elizabeth Central Hospital, by isolate and year

|  | Time period | | | | | | | |
| --- | --- | --- | --- | --- | --- | --- | --- | --- |
|  | 1998-2002 | | 2003-2007 | | 2008-2012 | | 2013-2017 | |
|  | N | % | N | % | N | % | N | % |
| **Gram-positives** |  |  |  |  |  |  |  |  |
| Group A Strep | 59 | 9.9 | 19 | 3.7 | 17 | 6.1 | 8 | 2.2 |
| Group B Strep | 71 | 11.9 | 71 | 13.7 | 31 | 11.1 | 30 | 8.2 |
| *Streptococcus pneumoniae* | 49 | 8.2 | 67 | 12.9 | 22 | 7.9 | 4 | 1.1 |
| *Staphylococcus aureus* | 60 | 10.1 | 77 | 14.8 | 52 | 18.7 | 56 | 15.4 |
| Other *Streptococcus* spp. | 22 | 3.7 | 12 | 2.3 | 9 | 3.2 | 3 | 0.8 |
| *Enterococcus* spp. | 7 | 1.2 | 10 | 1.9 | 10 | 3.6 | 25 | 6.9 |
| **Gram-negatives** |  |  |  |  |  |  |  |  |
| *Acinetobacter* spp. | 17 | 2.8 | 19 | 3.7 | 6 | 2.2 | 16 | 4.4 |
| *Citrobacter* spp. | 8 | 1.3 | 7 | 1.3 | 2 | 0.7 | 1 | 0.3 |
| *Enterobacter* spp. | 11 | 1.8 | 21 | 4.0 | 9 | 3.2 | 37 | 10.2 |
| *Escherichia coli* | 48 | 8.0 | 39 | 7.5 | 30 | 10.8 | 29 | 8.0 |
| *Haemophilus* *influenzae* type b | 14 | 2.3 | 10 | 1.9 | 3 | 1.1 | 3 | 0.8 |
| All *Haemophilus* spp. | 14 | 2.3 | 11 | 2.1 | 3 | 1.1 | 4 | 1.1 |
| *Klebsiella* spp. | 52 | 8.7 | 25 | 4.8 | 46 | 16.5 | 105 | 28.8 |
| *Neisseria* spp. | 1 | 0.2 | 1 | 0.2 | 1 | 0.4 | 1 | 0.3 |
| *Pseudomonas* spp. | 12 | 2.0 | 9 | 1.7 | 5 | 1.8 | 10 | 2.7 |
| *Salmonella* Typhi | 0 | 0 | 0 | 0 | 2 | 0.7 | 6 | 1.6 |
| NTS | 153 | 25.6 | 116 | 22.4 | 23 | 8.2 | 11 | 3.0 |
| *Serratia* spp. | 7 | 1.2 | 1 | 0.2 | 1 | 0.4 | 4 | 1.1 |
| Other *Enterobacteriaceae^a^* | 3 | 0.5 | 0 | 0 | 1 | 0.4 | 4 | 1.1 |
| Other Gram-negatives^b^ | 3 | 0.5 | 14 | 2.7 | 8 | 2.9 | 8 | 2.2 |
| **Fungus** |  |  |  |  |  |  |  |  |
| *Candida* | 0 | 0 | 0 | 0 | 1 | 0.4 | 1 | 0.3 |
| *Cryptococcus* | 0 | 0 | 0 | 0 | 0 | 0 | 1 | 0.3 |
| All pathogens^c^ | 597 | 100 | 519 | 100 | 279 | 100 | 364 | 100 |

NTS, nontyphoidal Salmonella

^a^Includes *Morganella* spp., *Pantoea* spp., *Raoultella* spp., *Shigella* spp., *Yersinia* spp.

^b^Includes *Aeromonas* spp., *Burkholderia* spp., Coliforms, *Edwardsiella* spp., Gram negative rods, *Moraxella* spp., *Pasteurella* spp., *Stenotrophomonas* spp., *Xanthomonas* spp.

^c^Excludes contaminants, including *Aerococcus* spp., alpha-hemolytic streptococci, *Bacillus* spp., *Clostridium* spp., coagulase-negative staphylococci, Corynebacteria, Diphtheroids, *Micrococcus* spp., *Rhizobium* spp.

**Supplementary Table 2C.** Bloodstream infections in children <7 days at Queen Elizabeth Central Hospital, by isolate and year

|  | Time period | | | | | | | |
| --- | --- | --- | --- | --- | --- | --- | --- | --- |
|  | 1998-2002 | | 2003-2007 | | 2008-2012 | | 2013-2017 | |
|  | N | % | N | % | N | % | N | % |
| **Gram-positives** |  |  |  |  |  |  |  |  |
| Group A Strep | 8 | 2.4 | 4 | 1.0 | 1 | 0.5 | 1 | 0.2 |
| Group B Strep | 40 | 12.1 | 39 | 9.9 | 6 | 3.3 | 13 | 2.8 |
| *Streptococcus pneumoniae* | 15 | 4.5 | 13 | 3.3 | 6 | 3.3 | 2 | 0.4 |
| *Staphylococcus aureus* | 62 | 18.8 | 61 | 15.5 | 35 | 19.2 | 90 | 19.3 |
| Other *Streptococcus* spp. | 14 | 4.2 | 24 | 6.1 | 9 | 4.9 | 22 | 4.7 |
| *Enterococcus* spp. | 18 | 5.5 | 33 | 8.4 | 23 | 12.6 | 51 | 10.9 |
| **Gram-negatives** |  |  |  |  |  |  |  |  |
| *Acinetobacter* spp. | 18 | 5.5 | 13 | 3.3 | 12 | 6.6 | 23 | 4.9 |
| *Citrobacter* spp. | 6 | 1.8 | 15 | 3.8 | 0 | 0 | 3 | 0.6 |
| *Enterobacter* spp. | 21 | 6.4 | 49 | 12.4 | 10 | 5.5 | 68 | 14.6 |
| *Escherichia coli* | 34 | 10.3 | 60 | 15.2 | 21 | 11.5 | 30 | 6.4 |
| *Haemophilus* spp. | 2 | 0.6 | 0 | 0 | 0 | 0 | 0 | 0 |
| *Klebsiella* spp. | 53 | 16.1 | 40 | 10.2 | 37 | 20.3 | 134 | 28.8 |
| *Neisseria* spp. | 0 | 0 | 0 | 0 | 1 | 0.5 | 0 | 0 |
| *Proteus* spp. | 8 | 2.4 | 3 | 0.8 | 0 | 0 | 0 | 0 |
| *Pseudomonas* spp. | 13 | 3.9 | 15 | 3.8 | 6 | 3.3 | 9 | 1.9 |
| *Salmonella* Typhi | 0 | 0 | 0 | 0 | 1 | 0.5 | 3 | 0.6 |
| NTS | 12 | 3.6 | 13 | 3.3 | 3 | 1.6 | 2 | 0.4 |
| *Serratia* spp. | 3 | 0.9 | 5 | 1.3 | 0 | 0 | 4 | 0.9 |
| Other *Enterobacteriaceae^a^* | 0 | 0 | 1 | 0.3 | 0 | 0 | 0 | 0 |
| Other Gram-negatives^b^ | 3 | 0.9 | 5 | 1.3 | 9 | 4.9 | 8 | 1.7 |
| **Fungus** |  |  |  |  |  |  |  |  |
| *Candida* | 0 | 0 | 1 | 0.3 | 1 | 0.5 | 1 | 0.2 |
| *Cryptococcus* | 0 | 0 | 0 | 0 | 1 | 0.5 | 0 | 0 |
| All pathogens^c^ | 330 | 100 | 394 | 100 | 182 | 100 | 466 | 100 |

NTS, nontyphoidal Salmonella

^a^Includes *Morganella* spp., *Raoultella* spp.

^b^Includes *Aeromonas* spp., *Burkholderia* spp., Coliforms, Gram negative rods, *Stenotrophomonas* spp.

^c^Excludes contaminants, including *Aerococcus* spp., *Clostridium* spp., Corynebacteria.
