# Supplementary material for: Emerging Resistance to Empiric Antimicrobial Regimens for Pediatric Bloodstream Infections in Malawi (1998–2017)
Source: Clin Infect Dis. 2018 Oct 1;69(1):61–8. doi: 10.1093/cid/ciy834 (PMC6579959; doi:10.1093/cid/ciy834)
Supplement: ciy834_suppl_Supplementary_Table_3 [file ciy834_suppl_supplementary_table_3.doc]

**Supplementary Table 3.** Antimicrobial resistance profiles of selected bloodstream pathogens for children ≤60 days, by period

| Pathogen | Time period | Antimicrobial resistance | | | | | | | | | | | | | | | |
| --- | --- | --- | --- | --- | --- | --- | --- | --- | --- | --- | --- | --- | --- | --- | --- | --- | --- |
|  |  | Ampicillin | | | Penicillin | | | Ceftriaxone | | Chloramphenicol | | Gentamicin | | Co-trimoxazole | | Ciprofloxacin | |
|  |  | N | % | N | | % | N | | % | N | % | N | % | N | % | N | % |
| *S. pneumoniae* | 1998-2002 | NT |  | 7/ 49 | | 14.3 | 0^a^/ 8 | | 0 | 9/ 50 | 18 | NT |  | 37/ 49 | 75.5 | NT |  |
|  | 2003-2007 | NT |  | 8/ 57 | | 14.0 | 0^a^/ 57 | | 0 | 10/ 57 | 17.5 | NT |  | 40/ 53 | 75.5 | NT |  |
|  | 2008-2012 | NT |  | 1/ 23 | | 4.3 | 0^a^/ 23 | | 0 | 3/ 23 | 13.0 | NT |  | 15/ 23 | 65.2 | NT |  |
|  | 2013-2017 | NT |  | 1/ 5 | | 20 | 0/ 5 | | 0 | 1/ 5 | 20 | NT |  | 5/ 5 | 100 | NT |  |
| *S. aureus* | 1998-2002 | NT |  | 114/ 116 | | 98.3 | NT | |  | 42/ 115 | 36.5 | 13^b^/ 115 | 11.3 | 53/ 117 | 45.3 | NT |  |
|  | 2003-2007 | NT |  | 117/ 125 | | 93.6 | NT | |  | 28/ 130 | 21.5 | 4^b^/ 106 | 3.8 | 48/ 129 | 37.2 | NT |  |
|  | 2008-2012 | NT |  | 16/ 19 | | 84.2 | NT | |  | 9/ 80 | 11.3 | 16^b^/ 78 | 20.5 | 32/ 79 | 40.5 | NT |  |
|  | 2013-2017 | NT |  | 5/ 28 | | 17.9 | NT | |  | 6/ 139 | 4.3 | 20^b^/ 139 | 14.4 | 50/ 159 | 31.4 | NT |  |
| *Enterococcus* spp. | 1998-2002 | 1/ 5 | 20 | 12/ 22 | | 54.5 | NT | |  | 11/ 23 | 47.8 | 16/ 17 | 94.1 | 15/ 24 | 62.5 | NT |  |
|  | 2003-2007 | 2/ 26 | 7.7 | 20/ 24 | | 83.3 | NT | |  | 26/ 42 | 61.9 | 4/ 4 | 100 | 20/ 42 | 47.6 | NT |  |
|  | 2008-2012 | 17/ 25 | 68 | NT | |  | NT | |  | 21/ 28 | 75 | 2/ 2 | 100 | 27/ 27 | 100 | NT |  |
|  | 2013-2017 | 54/ 72 | 75 | NT | |  | NT | |  | 56/ 71 | 78.9 | 1/ 1 | 100 | 53/ 71 | 74.6 | NT |  |
| *E. coli* | 1998-2002 | 59/ 73 | 80.8 | NT | |  | 2/ 14 | | 14.3 | 37/ 72 | 51.4 | 6/ 72 | 8.3 | 52/ 73 | 71.2 | 0/ 25 | 0 |
|  | 2003-2007 | 65/ 91 | 71.4 | NT | |  | 1/ 72 | | 1.4 | 35/ 93 | 37.6 | 17/ 91 | 18.7 | 71/ 92 | 77.2 | 1/ 94 | 1.1 |
|  | 2008-2012 | 36/ 47 | 76.6 | NT | |  | 13/ 42 | | 31.0 | 12/ 47 | 25.5 | 17/ 47 | 36.2 | 42/ 47 | 89.4 | 12/ 46 | 26.1 |
|  | 2013-2017 | 48/ 57 | 84.2 | NT | |  | 13/ 57 | | 22.8 | 22/ 57 | 38.6 | 8/ 57 | 14.0 | 56/ 57 | 98.2 | 10/ 57 | 17.5 |
| *Klebsiella* spp. | 1998-2002 | 98/ 98 | 100 | NT | |  | 2/ 12 | | 16.7 | 74/ 98 | 75.5 | 53/ 98 | 54.1 | 77/ 97 | 79.4 | 0/ 37 | 0 |
|  | 2003-2007 | 60/ 62 | 96.8 | NT | |  | 4/ 50 | | 8 | 41/ 61 | 67.2 | 35/ 62 | 56.5 | 38/ 58 | 65.5 | 1/ 62 | 1.6 |
|  | 2008-2012 | 76/ 77 | 98.7 | NT | |  | 58/ 67 | | 86.6 | 45/ 75 | 60 | 66/ 76 | 86.8 | 70/ 77 | 90.9 | 12/ 77 | 15.6 |
|  | 2013-2017 | 230/ 230 | 100 | NT | |  | 213/ 230 | | 92.6 | 116/ 229 | 50.7 | 214/ 228 | 93.9 | 220/ 230 | 95.7 | 68/ 230 | 29.6 |
| *Enterobacter* spp. | 1998-2002 | 23/ 29 | 79.3 | NT | |  | 2/ 2 | | 100 | 16/ 29 | 55.2 | 9/ 27 | 33.3 | 18/ 29 | 62.1 | 0/ 20 | 0 |
|  | 2003-2007 | 45/ 60 | 75 | NT | |  |  | | 100^c^ | 25/ 68 | 36.8 | 12/ 58 | 20.7 | 34/ 69 | 49.3 | 4/ 61 | 6.6 |
|  | 2008-2012 | 14/ 16 | 87.5 | NT | |  |  | | 100^c^ | 11/ 17 | 64.7 | 13/ 17 | 76.5 | 14/ 17 | 82.4 | 11/ 17 | 64.7 |
|  | 2013-2017 | 97/ 101 | 96.0 | NT | |  |  | | 100^c^ | 92/ 101 | 91.1 | 79/ 101 | 78.2 | 90/ 101 | 89.1 | 70/ 101 | 69.3 |
| *Acinetobacter* spp. | 1998-2002 | 23/ 30 | 76.7 | NT | |  | 2/ 2 | | 100 | 22/ 32 | 68.8 | 19/ 31 | 61.3 | 18/ 32 | 56.3 | 0/ 10 | 0 |
|  | 2003-2007 | 16/ 23 | 69.6 | NT | |  | 13/ 26 | | 50 | 20/ 27 | 74.1 | 9/ 27 | 33.3 | 17/ 25 | 68 | 2/ 27 | 7.4 |
|  | 2008-2012 | 13/ 15 | 86.7 | NT | |  | 10/ 15 | | 66.7 | 15/ 16 | 93.8 | 11/ 16 | 68.8 | 12/ 17 | 70.6 | 5/ 16 | 31.3 |
|  | 2013-2017 | 27/ 36 | 75 | NT | |  | 35/ 36 | | 97.2 | 31/ 36 | 86.1 | 16/ 36 | 44.4 | 22/ 36 | 61.1 | 17/ 36 | 47.2 |
| *Pseudomonas* spp. | 1998-2002 |  | NT^d^ | NT | |  | NT | |  |  | NT^d^ | 4/ 22 | 18.2 |  | NT^d^ | 0/ 15 | 0 |
|  | 2003-2007 |  | NT^d^ | NT | |  | NT | |  |  | NT^d^ | 13/ 24 | 54.2 |  | NT^d^ | 0/ 24 | 0 |
|  | 2008-2012 |  | NT^d^ | NT | |  | NT | |  |  | NT^d^ | 3/ 10 | 30 |  | NT^d^ | 3/ 10 | 30 |
|  | 2013-2017 |  | NT^d^ | NT | |  | NT | |  |  | NT^d^ | 6/ 19 | 31.6 |  | NT^d^ | 2/ 19 | 10.5 |
| *Salmonella* Typhi | 1998-2002 | 0 |  | 0 | |  | 0 | |  | 0 |  | 0 |  | 0 |  | 0 |  |
|  | 2003-2007 | 0 |  | 0 | |  | 0 | |  | 0 |  | 0 |  | 0 |  | 0 |  |
|  | 2008-2012 | 0/ 2 | 0 | NT | |  | 0/ 2 | | 0 | 0/ 2 | 0 | 2/ 2 | 100 | 0/ 2 | 0 | 0/ 2 | 0 |
|  | 2013-2017 | 6/ 7 | 85.7 | NT | |  | 0/ 7 | | 0 | 5/ 7 | 71.4 | 7/ 7 | 100 | 6/ 7 | 85.7 | 0/ 7 | 0 |
| NTS | 1998-2002 | 241/ 254 | 94.9 | NT | |  | 0/ 9 | | 0 | 143/ 254 | 56.3 |  | 100^e^ | 104/ 129 | 80.6 | 0/ 177 | 0 |
|  | 2003-2007 | 177/ 189 | 93.7 | NT | |  | 1/ 149 | | 0.7 | 168/ 191 | 88.0 |  | 100^e^ | 92/ 98 | 93.9 | 2/ 189 | 1.1 |
|  | 2008-2012 | 92/ 115 | 80 | NT | |  | 1/ 111 | | 0.9 | 82/ 115 | 71.3 |  | 100^e^ | 18/ 18 | 100 | 0/ 114 | 0 |
|  | 2013-2017 | 66/ 73 | 90.4 | NT | |  | 1/ 73 | | 1.4 | 42/ 73 | 57.5 | 73/ 73 | 100 | 8/ 9 | 88.9 | 0/ 73 | 0 |
| Other Enterobacteriaceae^f^ | 1998-2002 | 36/ 39 | 92.3 | NT | |  | 0/ 1 | | 0 | 30/ 39 | 76.9 | 13/ 40 | 32.5 | 25/ 40 | 62.5 | 1/ 25 | 4 |
|  | 2003-2007 | 27/ 34 | 79.4 | NT | |  | 5/ 26 | | 19.2 | 15/ 34 | 44.1 | 10/ 34 | 29.4 | 8/ 28 | 28.6 | 4/ 34 | 11.8 |
|  | 2008-2012 | 4/ 4 | 100 | NT | |  | 0/ 4 | | 0 | 0/ 4 | 0 | 1/ 4 | 25 | 1/ 4 | 25 | 1/ 4 | 25 |
|  | 2013-2017 | 16/ 19 | 84.2 | NT | |  | 9/ 18 | | 50 | 5/ 18 | 27.8 | 5/ 18 | 27.8 | 15/ 18 | 83.3 | 5/ 18 | 27.8 |
| Other Gram-negatives^g^ | 1998-2002 | 7/ 11 | 63.6 | 0/ 1 | | 0 | 0/ 11 | | 0 | 3/ 13 | 23.1 | 2/ 13 | 15.4 | 13/ 13 | 100 | 0 | 20 |
|  | 2003-2007 | 10/ 18 | 55.6 | 0/ 2 | | 0 | 0/ 17 | | 0 | 8/ 18 | 44.4 | 5/ 18 | 27.8 | 11/ 14 | 78.6 | 1/ 14 | 7.1 |
|  | 2008-2012 | 11/ 12 | 91.7 | 1/ 1 | | 100 | 8/ 15 | | 53.3 | 6/ 13 | 46.2 | 4/ 16 | 25 | 9/ 14 | 64.3 | 3/ 16 | 18.8 |
|  | 2013-2017 | 14/ 14 | 100 | 0/ 1 | | 0 | 9/ 19 | | 47.4 | 9/ 14 | 64.3 | 12/ 18 | 66.7 | 10/ 14 | 71.4 | 3/ 16 | 18.8 |

BSAC, British Society of Antimicrobial Chemotherapy; NT, not tested; NTS, nontyphoidal Salmonella

^a^Pneumococcal isolates initially reported as ceftriaxone-resistant were re-tested and found to be susceptible. Based on this, isolates not available for re-testing (4) were considered susceptible.

^b^Note that gentamicin should not be used alone for *S. aureus,* even if susceptible.

^c^All Enterobacter isolates have been reported resistant to ceftriaxone in line with BSAC guidance.

^d^All Pseudomonas isolates have been reported not tested as these pathogens are intrinsically resistant to ampicillin, chloramphenicol and co-trimoxazole.

^e^All Salmonella isolates have been reported resistant to gentamicin in line with BSAC guidance.

^f^Includes *Citrobacter* spp., Coliforms, *Escherichia* spp., *Kluyvera* spp., *Morganella* spp., *Pantoea* spp., *Proteus* spp., *Raoultella* spp., *Serratia* spp., *Shigella* spp., *Yersinia* .

^g^Includes *Aeromonas* spp., *Burkholderia* spp., *Edwardsiella* spp., Gram negative rods, *Haemophilus* spp., *Moraxella* spp., *Neisseria* spp., *Pasteurella* spp., *Stenotrophomonas* spp., *Xanthomonas* spp..
