# Supplementary material for: Emerging Resistance to Empiric Antimicrobial Regimens for Pediatric Bloodstream Infections in Malawi (1998–2017)
Source: Clin Infect Dis. 2018 Oct 1;69(1):61–8. doi: 10.1093/cid/ciy834 (PMC6579959; doi:10.1093/cid/ciy834)
Supplement: ciy834_suppl_Supplementary_Legends [file ciy834_suppl_supplementary_legends.docx]

**Supplementary Figure 1.** Blood culture rates at Queen Elizabeth Central Hospital for children ≤5 years, 1998-2017.

*Contaminants 1998-2002 were not recorded in complete form in the electronic database despite being in the books.

**Supplementary Table 1.** Admission statistics in Queen Elizabeth Central Hospital, by ward and year

**Supplementary Table 2.** Bloodstream infections at Queen Elizabeth Central Hospital, by isolate and year for children A) ≤60 days; B) 7-90 days; C) <7 days

**Supplementary Table 3.** Antimicrobial resistance profiles of selected bloodstream pathogens for children ≤60 days, by period
